# Supplementary material for: Contraction of basal filopodia controls periodic feather branching via Notch and FGF signaling
Source: Nat Commun. 2018 Apr 9;9:1345. doi: 10.1038/s41467-018-03801-z (PMC5890251; doi:10.1038/s41467-018-03801-z)
Supplement: Supplementary file 1 — Supplementary information [file 41467_2018_3801_MOESM1_ESM.pdf]

Supplementary Information

Contraction of basal filopodia controls periodic feather branching via Notch and FGF signalling

Cheng et al.

Supplementary Figure 1

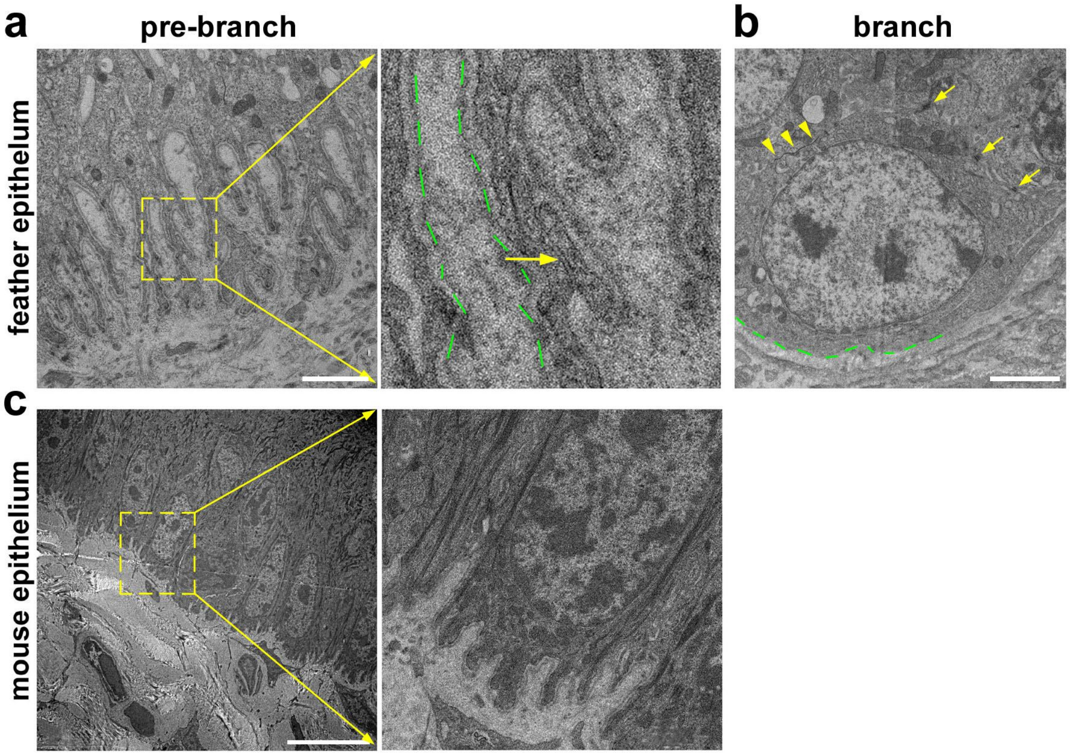

Supplementary Figure 1 | More TEM images showing the filopodia in basal keratinocytes and cell adhesion in the branched feather epithelium – related to Fig. 2. (**a**) Higher magnification view showing two adjacent cells are separated inside a fused filopodium (arrow). (**b**) An outer barb cell connects with its neighbor cells via tight junctions (arrow heads) and desmosomes (yellow arrows). Dashed lines indicate the basal lamina. (**c**) For comparison, basal keratinocytes in the mouse footpad skin also have filopodia but are much shorter. Representative images from five experiments are shown. Bar = 2μm in **a**, **b**, 10μm in **c**.

Supplementary Figure 2

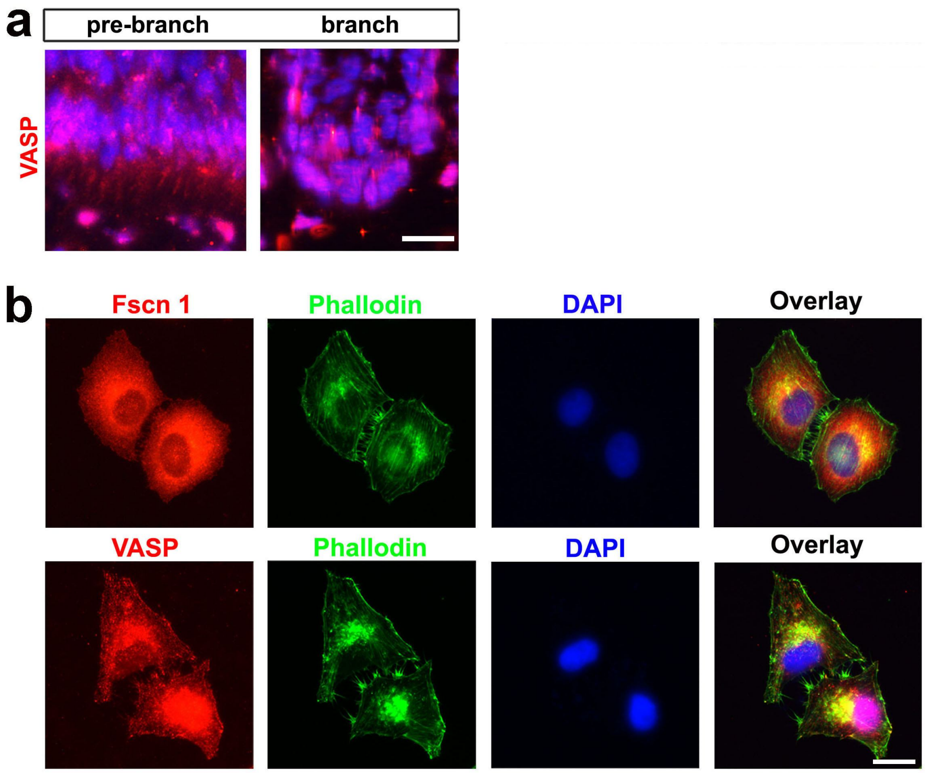

Supplementary Figure 2 | Additional marker analysis in the feather filopodia and verification of the antibodies – related to Fig. 2. (a) VASP was localized in the filopodia of basal keratinocytes. (**b)** Fscn1 and Vasp were localized in the filopodia in HeLa cells. Representative images from three experiments are shown. Bar = 10μm.

Supplementary Figure 3

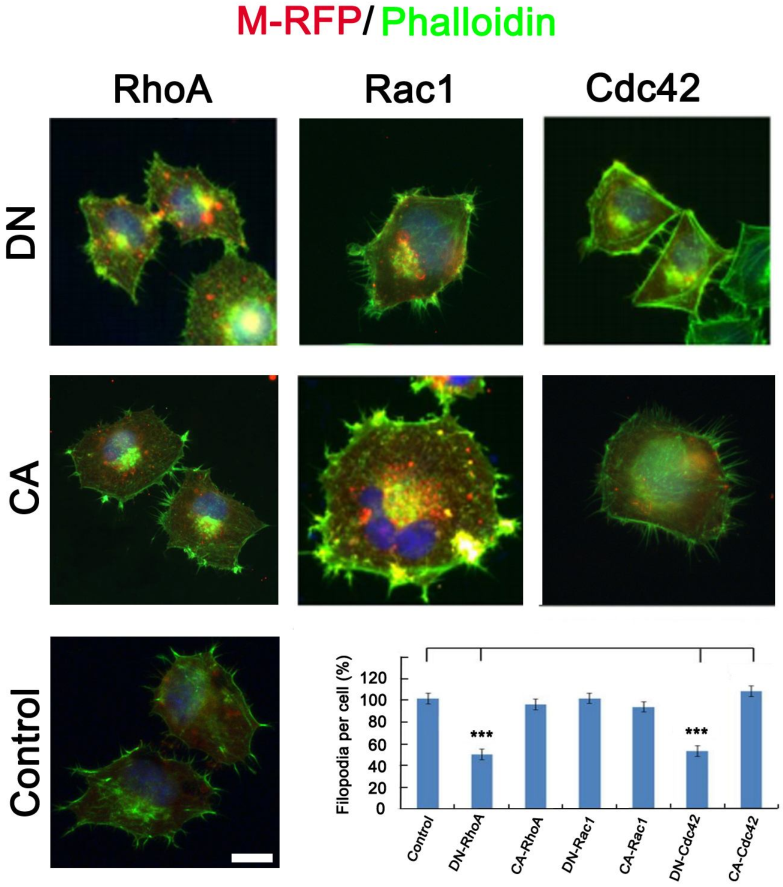

Supplementary Figure 3 | Rho GTPases regulate filopodia in cell culture. DN-*RhoA* and DN-*Cdc42* inhibited filopodia formation in HeLa cells. Genes were cloned into the lentiviral vector pLVX-ZsGreen and electroporated into HeLa cells. Cell membrane was shown by co-transfection with a membrane RFP plasmid (M-RFP). Filopodia were stained by FITC-phalloidin and counted for each cell. N=20 cells were counted and representative images are shown. Values are means ± s.e.m. ***, $p < 0.001$ by *t*-test. Bar = 10μm.

Supplementary Figure 4

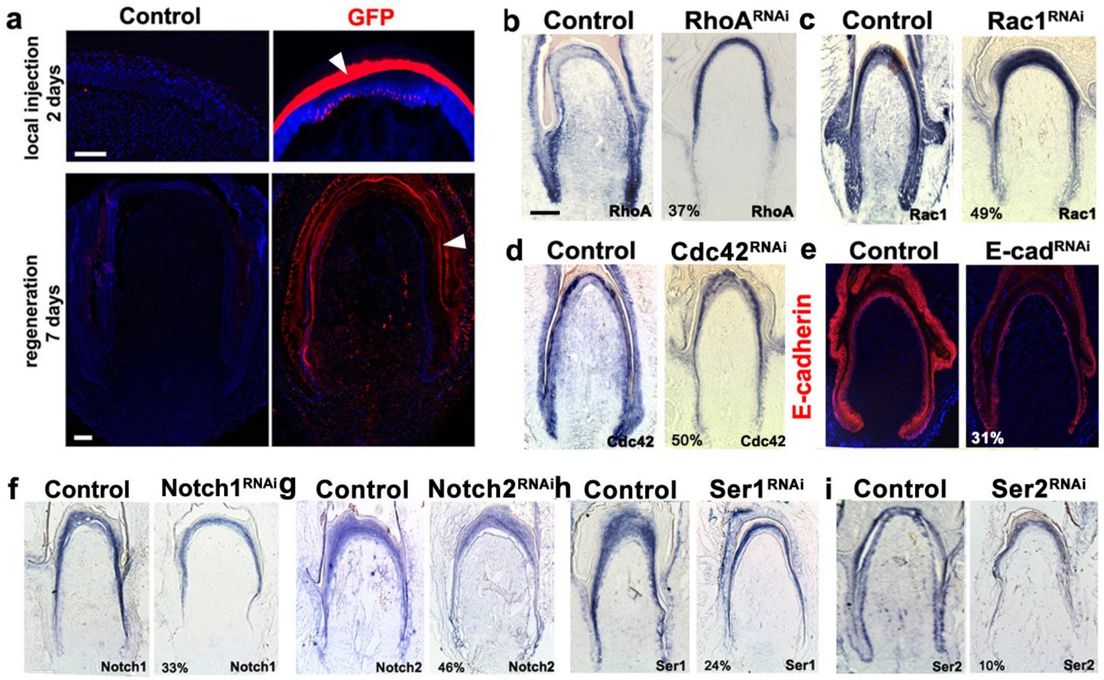

Supplementary Figure 4 | Validation of virus expression and RNAi knockdown efficiency in the feather follicle. (**a**) Virus expression was examined by a GFP antibody, which is carried by the viral vector under a CMV promoter. Both local injection (samples collected 2 days post injection) and feather regeneration (samples collected 7 days post injection) were examined. Arrowheads indicated the keratinized feather sheath which showed background fluorescence. (**b-d & f-i**) In situ hybridization, and (**e**) E-Cadherin antibody staining (red) showing lentiviral-mediated RNAi knockdown in the feather follicle. Feathers were regenerated for 4 days before sample collection. Some background signal was found in the keratinized feather sheath. The numbers indicated the relative expression levels as compared with control follicles (quantified by qRT-PCR). Representative images from three repeats are shown. Bar = 100μm.

Supplementary Figure 5

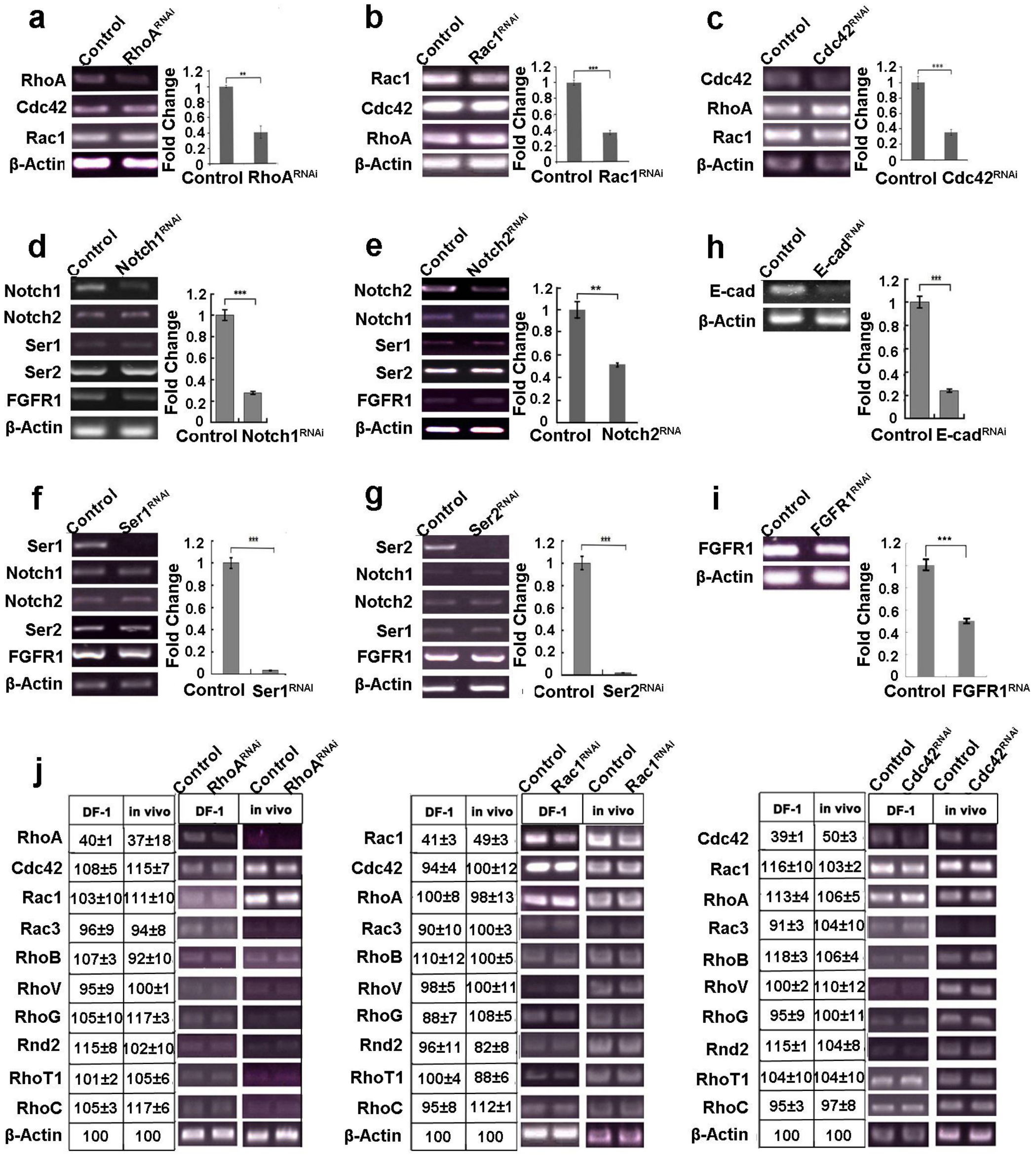

Supplementary Figure 5 | Specificity and efficiency of RNAi knockdown. (a-i) RNAi constructs were electroporated into DF-1 cells. A scramble control was used. Cells were lysed 48 hours post electroporation, and total RNAs were extracted. RT-PCR and qRT-PCR were performed. (**j**) Specificity of RNAi knockdown. Feather follicles were collected individually at 4 days post-infection for RNA extraction and quantification. Results from *in vivo* experiments were compared with those from DF-1 cells (percentage value of control). Values are means ± s.e.m. from three independent experiments. *, p<0.1; **, p<0.01; ***, p<0.001 by t-test.

Supplementary Figure 6

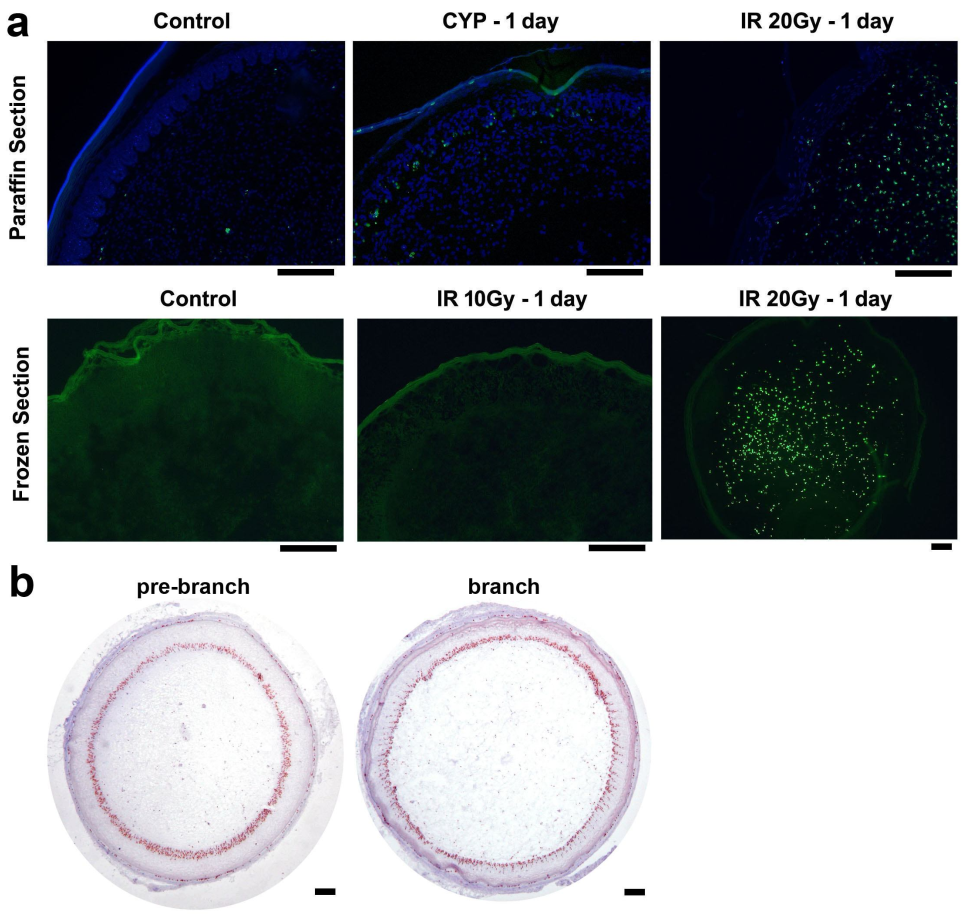

Supplementary Figure 6 | Lack of pre-patterned cell proliferation or apoptosis during feather branching. (a) TUNEL analysis was performed on feather samples treated by various methods. Normal feathers don't show positive TUNEL signal before or immediately after branching. For positive controls, treatment by the chemotherapeutic agent cyclophosphamide (CYP, 150 mg kg^{-1}) or ionizing radiation (IR) induced extensive TUNEL positive cells in the feather follicle, as demonstrated previously[1, 2]. (**b)** BrdU staining showed no pre-patterned cell proliferation in feather branching. BrdU (100mg kg^{-1} in PBS) was i.p. injected 2 hours before sample collection. Bar = 100μm.

Supplementary Figure 7

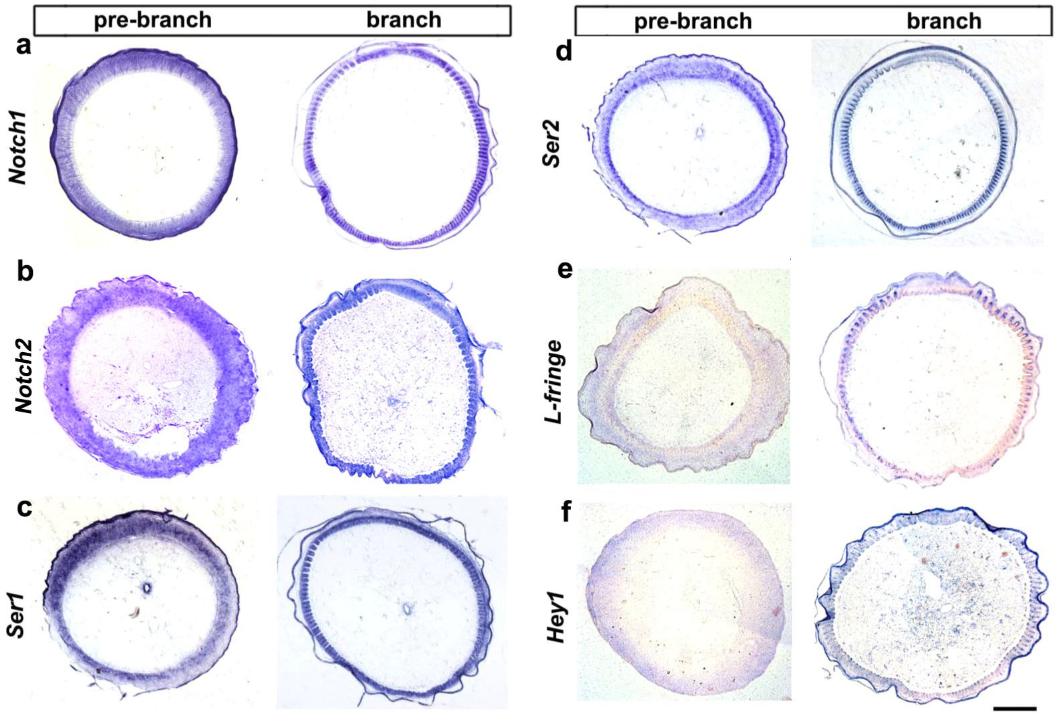

Supplementary Figure 7 | Additional gene expression analysis in the feather follicle – related to Fig. 5. In situ hybridization was performed in cross sections before and after branching to show the gene expression patterns. (**a**) *Notch1*; (**b**) *Notch2*; (**c**) *Serrate1*; (**d**) *Serrate2*; (**e**) *L-fringe*; (**f**) *Hey1*. In the anterior-posterior axis, there is no graded expression of Notch signalling components (*Notch1*, *Notch2*, *Ser1*, *Ser2*). *L-fringe* and *Hey1* showed a *de novo* expression pattern in the marginal plate during feather branching. Anterior is where the rachis locates. Bar = 200μm.

Supplementary Figure 8

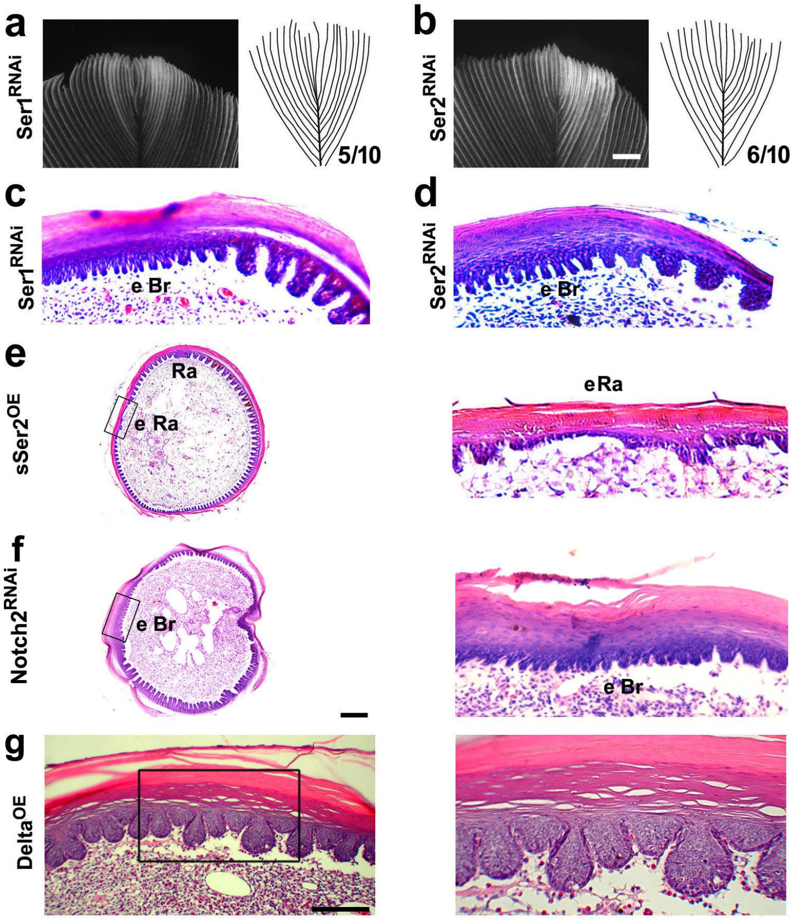

Supplementary Figure 8 | Additional phenotype analysis – related to Fig. 5. (a,b) RNAi-*Ser1/2* reduced feather rachis formation. (**c-f)** Local injection of lentivirus carrying the transgenes. RNAi-*Ser1/2* and *Notch2* induced ectopic branching in the rachis (Ra), whereas a secretory form of Ser2 induced ectopic rachis (eRa). OE, overexpression. (**g**) RCAS-mediated *Delta* overexpression induced irregular branching in the feather follicle. The boxed areas in **e,f,g** are enlarged. Each experiment was repeated at least five times. Bar = 100μm.

Supplementary Figure 9

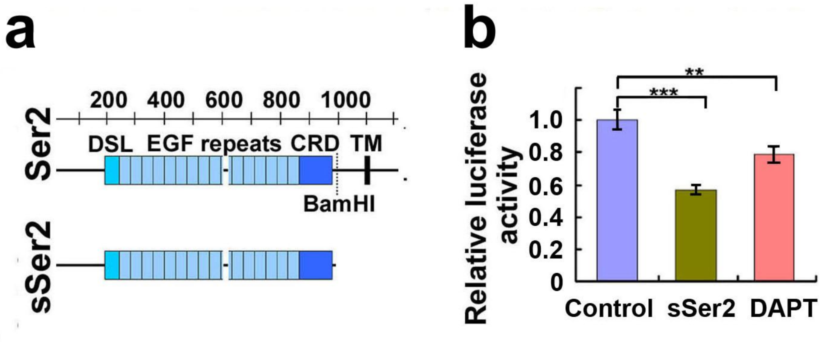

Supplementary Figure 9 | Construction of a secretory form of Serrate2. (**a**) Full-length human *SERRATE2* gene contains a BamHI site before the transmembrane domain, which was used to construct the secretory form of Serrate2 (sSer2). (**b**) Over expression of sSer2 inhibited the activity of a 6XCSL Notch reporter in 293T cells. The lentiviral vector pLL3.7 was used as control, and the Notch inhibitor DAPT was used as a positive control. sSer2 (300ng/1μg total plasmids per each well in 24-well plate transfection) inhibited Notch reporter activity more effective than DAPT (46μM) under the experimental condition. Each experimental condition was repeated three times. **, p<0.01; ***, p<0.001 by t-test.

Supplementary Figure 10

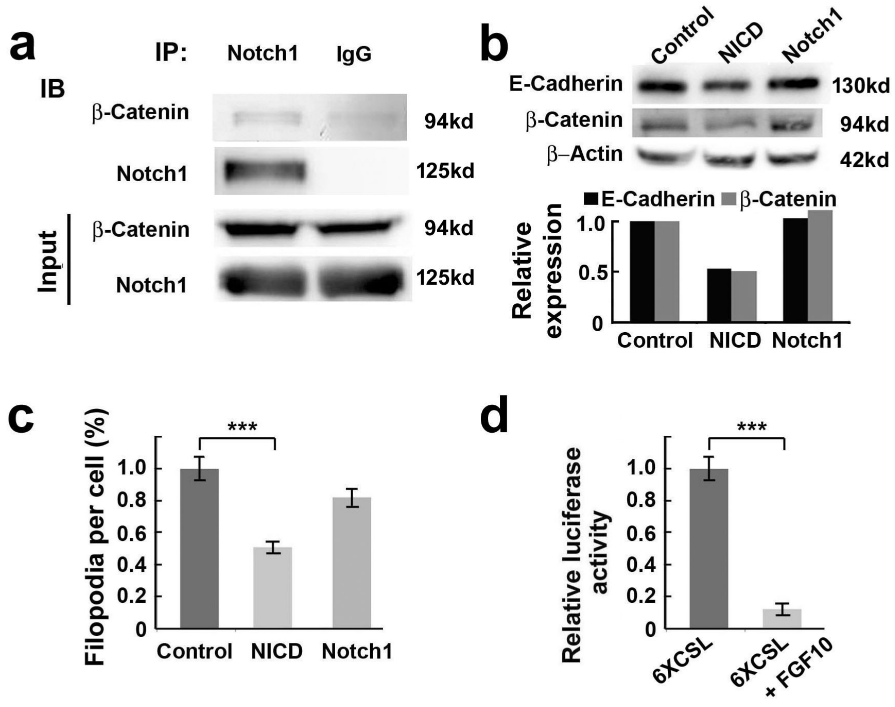

Supplementary Figure 10 | Notch activation impacts several aspects of cell behaviour in vitro. (**a**) β-Catenin co-immunoprecipitated with Notch1 (myc-tagged) in 293T cells. Normal rabbit IgG was used as a control for the immunoprecipitation. (**b**) Activation of Notch signaling reduced the expression of E-Cadherin and β-Catenin. MCF7 cells were electroporated with the control vector (pEGFP-N1), *NICD* or the full-length *Notch1* expression plasmids, and lysed for Western blot analysis. The original gel images are in Supplementary Fig. 12. (**c**) Notch activation reduced the filopodia count in HeLa cells (N=20). (**d**) FGF10 inhibited Notch activation in 293T cells, as measured by the 6XCSL reporter assay. *FGF10* was transfected at 400ng/1μg total plasmids per each well in 24-well plate. Each experiment was repeated three times and representative results are shown. ***, p<0.001 by t-test.

Supplementary Figure 11

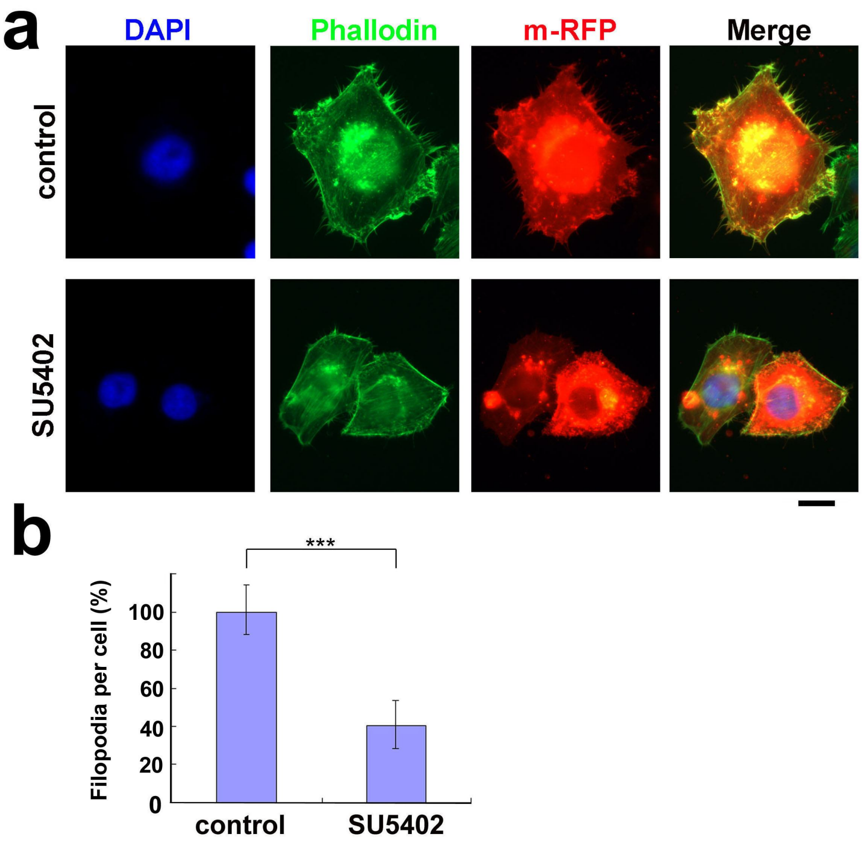

Supplementary Figure 11 | FGF signaling regulates filopodia in cell culture. (a) HeLa cells were transfected with membrane-RFP (m-RFP), treated with SU5402 (18μM 2 hours), fixed in 4% PFA, and stained with FITC-Phalloidin (1μg/ml). Representative images of 20 cells are shown. Bar = 10μm. (**b)** SU5402 treatment reduced the filopodia count. N=20; ***, p<0.001 by t-test.

Supplementary Figure 12

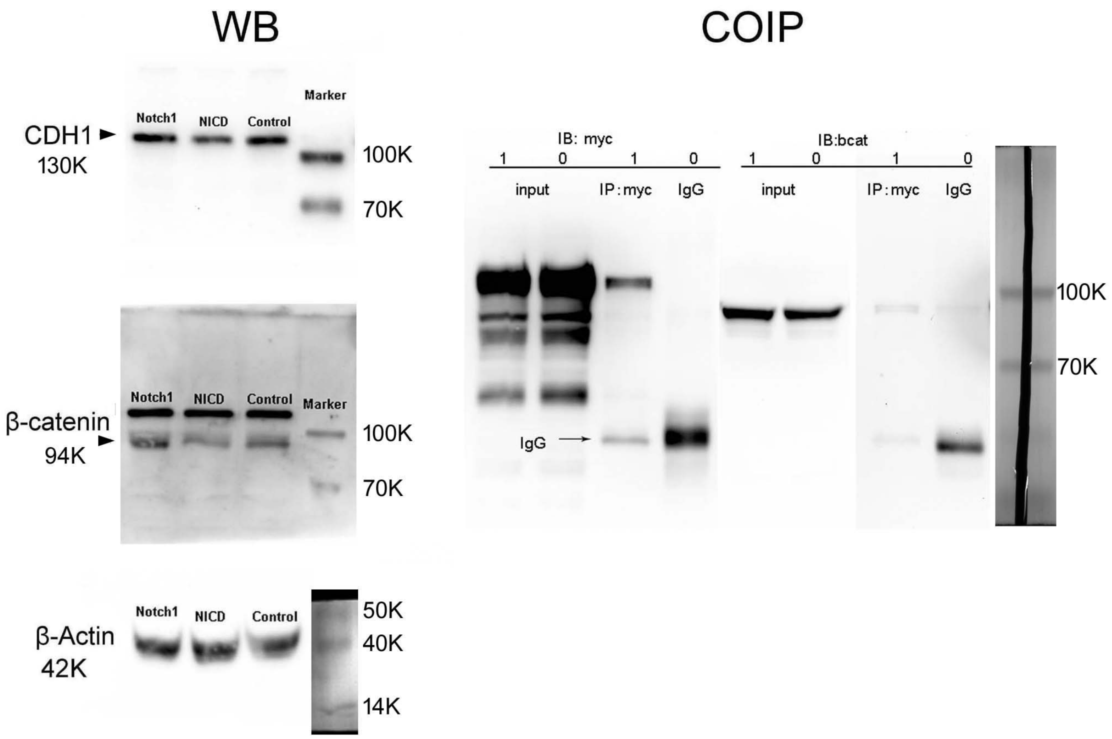

Supplementary Figure 12 | Uncropped gel images. For Western blot analysis, the blot was cut along the 50kd line. The upper blot was stained for Cdh1, followed by β-Catenin. The lower blot was stained for β-Actin.

Supplementary Note 1

Detailed coding information to execute the ACM algorithm is provided below, which is implemented in MATLAB R2012a under the Windows XP system.

```
for k1=1: 1000
    u=NeumannBoundCond(u);        %Initial contour
    K=curvature_central(u);       %Curvature
    DrcU=(epsilon/pi)./(epsilon^2.+u.^2);      %The Dirac function
    [f1, f2] = localBinaryFit(Img, u, KI, KONE, Ksigma, epsilon); %Last two term in Eq.(ii)
    [C1, C2]= binaryfit(Img,u,epsilon);     %First two terms in Eq.(ii)
    s1=lambda1.*f1.^2-lambda2.*f2.^2;
    s2=lambda1.*f1-lambda2.*f2;
    GdataForce=-eta1.*(Img-C1).^2+eta2.*(Img-C2).^2;

LdataForce=(lambda1-lambda2)*KONE.*Img.*Img+conv2(s1,Ksigma,'same')-2.*Img.*conv2(s
2,Ksigma,'same');
    G=DrcU.*GdataForce;
    A=-DrcU.*LdataForce;
    P=mu*(4*del2(u)-K);
    L=nu.*DrcU.*K.*g;
    S=DrcU.*g;
    u=u+timestep*(A+P+G+S+L);      %Eq.(iv)
end

function [f1, f2]= localBinaryFit(Img, u, KI, KONE, Ksigma, epsilon)
% compute f1 and f2
Hu= 0.5*(1+ (2/pi)*atan(u./epsilon));
I=Hu.*Img;
c1=conv2(Hu,Ksigma,'same');
c2=conv2(I,Ksigma,'same');
f1=c2./(c1);
f2=(KI-c2)./(KONE-c1);

function [C1,C2]= binaryfit(Img,u,epsilon)
Hu= 0.5*(1+ (2/pi)*atan(u./epsilon));
I=Hu.*Img;
numer_1=sum(I(:));
denom_1=sum(Hu(:));
C1= numer_1/denom_1;
I2=(1-Hu).*Img;
numer_2=sum(I2(:));
```

```
I3=1-Hu;
denom_2=sum(I3(:));
C2 = numer_2/denom_2;

function g = NeumannBoundCond(f)
% Neumann boundary condition
[nrow,ncol] = size(f);
g = f;
g([1 nrow],[1 ncol]) = g([3 nrow-2],[3 ncol-2]);
g([1 nrow],2:end-1) = g([3 nrow-2],2:end-1);
g(2:end-1,[1 ncol]) = g(2:end-1,[3 ncol-2]);

function k = curvature_central(u)
% compute curvature
[ux,uy] = gradient(u);
normDu = sqrt(ux.^2+uy.^2+1e-10);
Nx = ux./normDu;
Ny = uy./normDu;
[nxx,junk] = gradient(Nx);
[junk,nyy] = gradient(Ny);
k = nxx+nyy;
```

Supplementary Table 1 | Relative expression levels of Rho GTPase family members and Notch pathway genes in the feather follicle.

Gene	FPKM	Gene	FPKM
Actb	1221	*Gapdh*	1596
RhoA	461	*Cdh1*	125
Cdc42	190	*Ctnnb1*	1103
Rac1	112	*Notch1*	11
Rac3	68	*Notch2*	16
RhoB	63	*Serrate1*	14
RhoV	42	*Serrate2*	59
RhoG	28	*L-Fringe*	14
Rnd2	18	*R-Fringe*	21
RhoT1	18	*Hey1*	22
RhoC	10		

Supplementary Table 2 | Primers and target sequences for RNAi.

Name	Gene	Sequence (5'-3')		Product size	Location
qPCR-β-Actin	NM_205518.1	ss:<br>as:	CTGACGGACTACCTCATGAA<br>CCTCTCATTGCCAATGGTGA	210bp	nt621-830
qPCR-RhoA	NM_204704.1	ss:<br>as:	TGGATGGAAAGCAGGTGGAG<br>AGGCACGTTGGGACAGAAAT	191bp	nt143-333
qPCR-Cdc42	NM_205048.1	ss:<br>as:	TGTGGGTGATGGTGCTGTTG<br>GTGGATAGCTGAGGGGTCGT	197bp	nt24-220
qPCR-Rac1	NM_205017.1	ss:<br>as:	ATCAAGTGTGTGGTGGTGGG<br>GGTAGGAGAGTGGGCGTAGT	208bp	nt10-217
qPCR-Rac3	NM_205016.1	ss:<br>as:	TCCCATCACCTACCCCCAAG<br>TTTTGCCAGGCTTCTTCACG	158bp	nt405-562
qPCR-RhoB	NM_204909.1	ss:<br>as:	GAACTACGTGGCCGACATCG<br>GACTTCGGGCACCCACTTCT	190bp	nt120-309
qPCR-RhoC	NM_001029849.1	ss:<br>as:	ACATCGCCGACATTGAGGTG<br>CTCCGGGGTCCACTTCTCAG	182bp	nt125-306
qPCR-RhoV	XM_426425.4	ss:<br>as:	AGGCAGACTTGCGGGATGAT<br>GCTTTGTGCTCAACACCGCT	196bp	nt419-614
qPCR-RhoG	XM_015280950.1	ss:<br>as:	TCTGCTACACCACCAACGCC<br>TCTGGGGGTAGGAGAGCGTC	162bp	nt62-223
qPCR-Rnd2	NM_001252123.1	ss:<br>as:	GTCCGTCCATTGGCATACCC<br>CCGCAACGTGTTCAGGTCTG	183bp	nt211-393

qPCR-RhoT1	NM_001006208.1	ss:<br>as:	CTTAGATGTACAGCGGTGCC<br>CAGCCTTTCATTCCAACAAC	174bp	nt1116-1289
qPCR-FGF2	NM_205433.1	ss:<br>as:	GCAAACCGCTTTCTGGCTAT<br>GCTTTCTGTCCAGGTCCAGT	200bp	nt341-540
qPCR-FGF7	NM_001012525.1	ss:<br>as:	AACAAGTCAGGAAGACTCTATGG<br>TGATTAGGATATTGCCAGAGG	225bp	nt438-662
qPCR-FGF8	NM_001012767.1	ss:<br>as:	CGGGGTTCTACATCTGCATG<br>GCGGTTGAAGGGGTAGTTGA	290bp	nt311-600
qPCR-FGF10	NM_204696.1	ss:<br>as:	GGATACTGACAAATGGTGCC<br>GTCTCCTTGGAGGTGATTGT	230bp	nt251-480
qPCR-FGF12	NM_204888.1	ss:<br>as:	AGCGTGGTTCCTGGGACTCA<br>ATGGAGGGAGGGAGGGTGTTT	246bp	nt559-804
qPCR-FGFR1	NM_205510.1	ss:<br>as:	CTGGACCTATCCCGAGAAGA<br>CCCGTATTTGTTCTCCACGA	250bp	nt511-760
qPCR-Notch1	NM_001030295.1	ss:<br>as:	CAACTGCAAGCAGGACGTGAA<br>TGGCAGACAGGTGCAGTCGTA	223bp	nt549--771
qPCR-Notch2	NM_001252033.1	ss:<br>as:	CCCTCTCCCCAGTCATTTGT<br>ACTCGGTGGAAACATTGGCT	480bp	nt6173-6652
qPCR-Serrate1	XM_415035.5	ss:<br>as:	CGTGTGACTTGCGCAGAACATT<br>TCTTTGTCACAGAGCTGACCAC	338bp	nt694--1031
qPCR-Serrate2	XM_004936419.2	ss:<br>as:	GATCGTCATCCCGTTCCAGT<br>ATCCAACCTTCCATGCAGGCT	321bp	nt576--896
qPCR-E-cadherin	NM_001039258.2	ss:<br>as:	GGACTGTTGAGATAAGGGGC<br>ACTCACACACCTGGGCTTTG	144bp	nt2031--2174
Probe-Notch1	NM_001030295.1	ss:<br>as:	CAACTGCAAGCAGGACGTGAA<br>TGGCAGACAGGTGCAGTCGTA	223bp	nt549--771
Probe-Notch2	NM_001252033.1	ss:<br>as:	CCCTCTCCCCAGTCATTTGT<br>ACTCGGTGGAAACATTGGCT	480bp	nt6173-6652

Probe-Serrate1	XM_415035.5	ss:<br>as:	CCTTGCAGCTTCGGATCCAAAT<br>TGGGTTCCACAGTAGTTCA	638bp	nt415-1052
Probe-Serrate2	XM_004936419.2	ss:<br>as:	GGAGATACATTCCGCTGTTCG<br>AGAGGTTCAGAGGCACTGCAT	671bp	nt2278-2948
Probe-L-fringe	NM_204948.1	ss:<br>as:	CCAAGAAGTTCCACAAAGCG<br>TGCAATACCACAGCAACGAG	896bp	nt385-1280
Probe-Hey1	XM_015282862.1	ss:<br>as:	GCAACGCCTTTGGACATC<br>GCAGGCTTCCCCACCCTTA	408bp	nt749-1156
Probe-RhoA	NM_204704.1	ss:<br>as:	AGTGAGGGTTCTGTGGTTTC<br>AACAGCAAGAAGTTCACAGG	509bp	nt1153-1661
Probe-Rac1	NM_205017.1	ss:<br>as:	CTTTTCCCTTGTGAGTCCTG<br>ATGTGATGCTCCATTGTTCT	397bp	nt261-657
Probe-Cdc42	NM_205048.1	ss:<br>as:	CCCTTCTGCAAAGCTGGTGT<br>AGCACCAGCCTGGGACATCT	484bp	nt830-1313
Probe-FGF2	NM_205433.1	ss:<br>as:	GCACTTCAAGGACCCCAAG<br>GCTTTCTGTCCAGGTCCAGT	363bp	nt178-540
Probe-FGF10	NM_204696.1	ss:<br>as:	CAATGTGCAAATGGATACTGAC<br>TCTATGACATTACTACCATTGG	642bp	nt239-880
RNAi-Notch1	NM_001030295.1		GATTCGCAGCTGCTACCCA		nt7396-7414
RNAi-Notch2	NM_001252033.1		GCACGCTTCGTTAAGCAAG		nt6277-6295
RNAi-Serrate1	XM_415035.5		CTCTACTAATCCCGATCGC		nt576-594
RNAi-Serrate2	XM_004936419.2		TACTGAACCGGACGAATAT		nt1164-1182
RNAi-FGFR1	NM_205510.1		CTCTACTAATCCCGATCGC		nt147-164
RNAi-E-cadherin	NM_001039258.		GGGACAACGTCTACAACTA		nt2358-2376
RNAi-RhoA	NM_204704.1		GACTACGATCGACTTAGAC		nt241-259
RNAi-Rac1	NM_205017.1		GAGATAGGTGCAGTGAAAT		nt460-478
RNAi-Cdc42	NM_205048.1		GAACTCCTAGGGCTGTTCT		nt1213-1231

Supplementary references

1. Chen, X. *et al*. Dissecting the molecular mechanism of ionizing radiation-induced tissue damage in the feather follicle. *Plos One* **9**, e89234 (2014).

2. Xie, G. *et al*. Testing chemotherapeutic agents in the feather follicle identifies a selective blockade of cell proliferation and a key role for sonic hedgehog signaling in chemotherapy-induced tissue damage. *J. Invest. Dermatol.* **135**, 690-700 (2015).
